# Targeting the reactive intermediate in polysaccharide monooxygenases

**DOI:** 10.1007/s00775-017-1480-1

**Published:** 2017-07-11

**Authors:** Erik D. Hedegård, Ulf Ryde

**Affiliations:** Department of Chemistry, Chemical Centre, Lund University, Sölvegatan 39, Lund, Sweden

**Keywords:** Lytic polysaccharide monooxygenase, Density functional theory, Reaction mechanism, Computational chemistry

## Abstract

Lytic polysaccharide monooxygenases (LPMOs) are copper metalloenzymes that can enhance polysaccharide depolymerization through an oxidative mechanism, making them interesting for the production of biofuel from cellulose. However, the details of this activation are unknown; in particular, the nature of the intermediate that attacks the glycoside C–H bond in the polysaccharide is not known, and a number of different species have been suggested. The homolytic bond-dissociation energy (BDE) has often been used as a descriptor for the bond-activation power, especially for inorganic model complexes. We have employed quantum-chemical cluster calculations to estimate the BDE for a number of possible LPMO intermediates to bridge the gap between model complexes and the actual LPMO active site. The calculated BDEs suggest that the reactive intermediate is either a Cu(II)–oxyl, a Cu(III)–oxyl, or a Cu(III)–hydroxide, which indicate that O–O bond breaking occurs before the C–H activation step.

**Electronic supplementary material:**

The online version of this article (doi:10.1007/s00775-017-1480-1) contains supplementary material, which is available to authorized users.

## Introduction

**Scheme 1:**
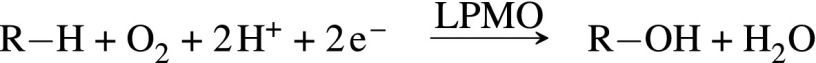
Reaction catalyzed by LPMO

The class of enzymes called lytic polysaccharide monooxygenases (LPMOs) [1–10] has recently attracted considerable attention due to their ability to enhance polysaccharide depolymerization, thereby providing a route to efficient conversion of polysaccharides into smaller sugars [11–16]. The enhancement proceeds by an oxidative mechanism in which the otherwise unreactive C–H bonds in the linkage between the sugar units of the polysaccharides are activated. The ability of the LPMOs to activate inert C–H bonds has its origin in an active site that contains a copper ion [1–10] and performs a reaction that involves O$_{2}$ and two reduction steps (cf. Scheme 1).

**Fig. 1:**
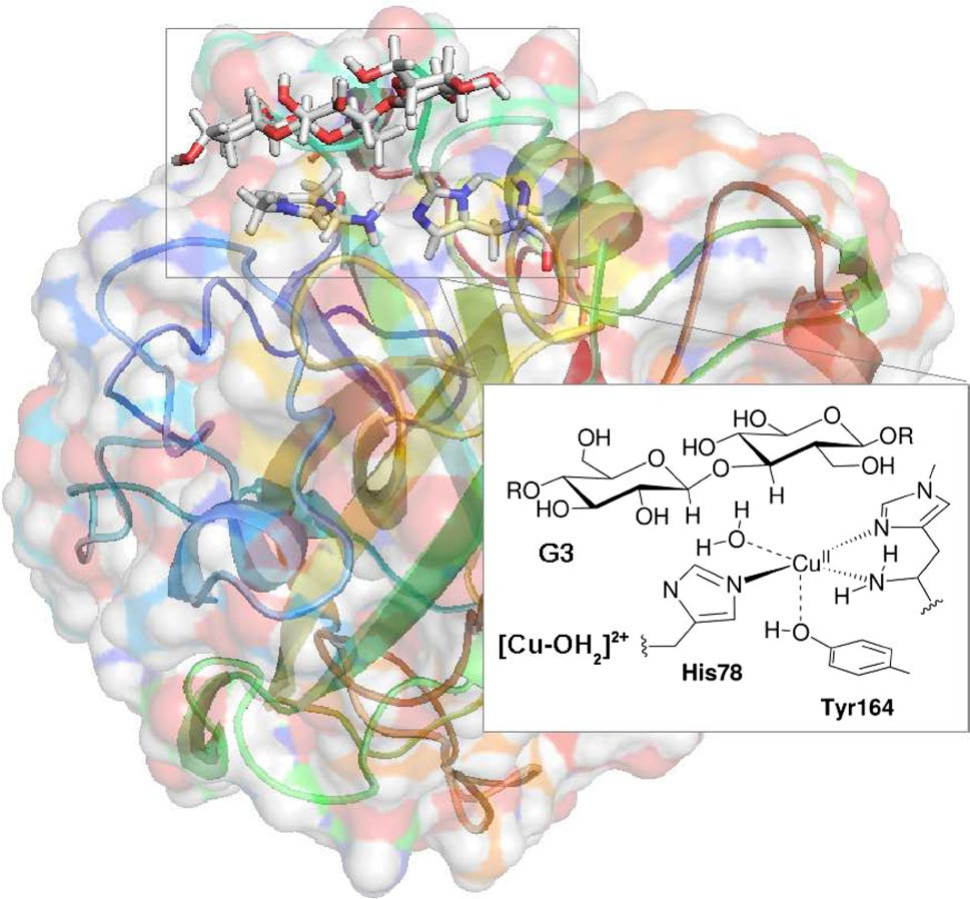
Active site of an LPMOs in the resting state, [Cu–OH$_{2}$]$^{2+}$ with a bound trisaccharide substrate (G3). Residue numbers refer to the enzyme from *Lentinus similis* [20]

Several crystal structures of LMPOs have been presented [1, 6, 12, 17–19], including a recent resting-state structure with a bound substrate [20], which is shown in Fig. 1. The coordination geometry in the oxidised Cu(II) intermediate is octahedral with a Jahn–Teller distortion. The equatorial ligand sphere consists of three nitrogen donor atoms in a so-called *histidine brace* moiety [1]. The histidine brace is comprised of the N$^{\delta 1}$ atom of a monodentate histidine and the (bidentate) N-terminal histidine residue, coordinating with both the amino-terminal N atom and the N$^{\delta 1}$ atom of the imidazole ring. The fourth equatorial ligand is a solvent molecule.

Another solvent molecule and a tyrosine phenol group are axial ligands. The axial water molecule dissociates upon substrate binding and is thus not present in Fig. 1.

**Scheme 2:**
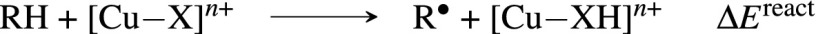
Hydrogen abstraction by the LPMO active intermediate

**Fig. 2:**
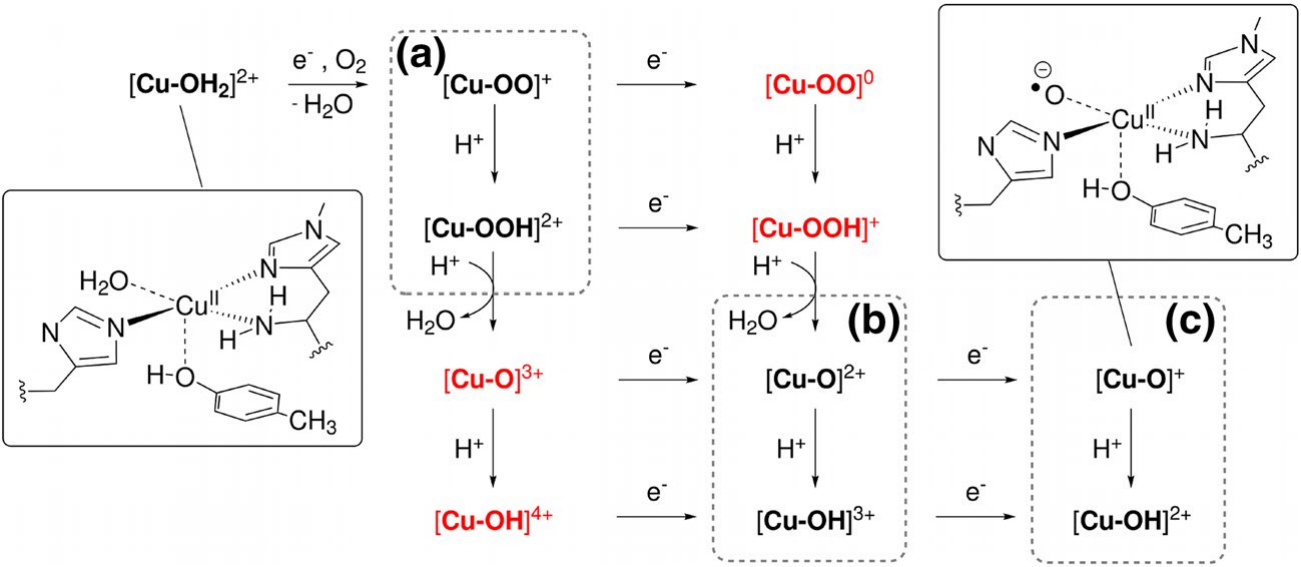
Overview over possible intermediates responsible for the C–H activation in LPMOs and their relation in terms of oxidation/reduction and protonation/deprotonation reactions. The product of the hydrogen-abstraction reaction in Scheme 2 is equivalent with the addition of an electron and a proton to the reactant, leading to the species one step along the diagonal down and right in the figure. Intermediates in dashed boxes **a**–**c** are considered in this study. The *insets* show Lewis structures of two of the intermediates

The reaction cycle of LPMOs is not fully understood. The first electron in Scheme 1 is used to reduce the active site from Cu(II) to Cu(I). This reduction is most likely associated with a dissociation of the equatorial water molecule [20, 21]. Thereafter, dioxygen binds, forming a Cu(II)–superoxide complex, [Cu–OO]$^{+}$, as is shown in the upper left corner of Fig. 2 (in the following, we will denote all species with only the atoms in the O$_{2}$-derived ligand and the net charge, to avoid speculating about the formal oxidation states of the Cu ion and the ligand, which often are less clear). The substrate binds either before, during, or after this process. A computational study and some X-ray structures [17] have suggested that O$_{2}^{-}$ binds *trans* to the tyrosine ligand [22], but more recent results [21, 23] favor the equatorial position. This is also supported by a recent X-ray structure of a trapped intermediate, showing O$_{2}^{-}$ (or O$_{2}^{2-}$) in the equatorial position [24]. At some point after this step, the activation of C–H bonds occurs, but the mechanism for this step is more unclear. A likely mechanism is that a yet unknown intermediate, [Cu–X]$^{n+}$, performs a hydrogen-atom abstraction from the substrate, as shown in Scheme 2.

It should then be followed by the rebound of a OH$^{\cdot }$ radical (from [Cu–XH]$^{n+}$) to the R$^{\cdot }$ radical.

Figure 2 shows a number of putative species that could be responsible for the C–H abstraction and their relations in terms of oxidation/reduction and protonation/deprotonation steps (see also Ref. [25]). After the formation of [Cu–OO]^+^, the overall stoichiometry of Scheme 1 indicates the addition of an additional electron and two protons, but the order of these steps in relation to the cleavage of the O–O bond is not known. The reactive species in LPMO could either be the superoxide complex, [Cu–OO]$^{+}$ (or [Cu–OOH]$^{2+}$), but some suggestions have instead involved an oxyl species [2, 3, 15, 17, 25]. Figure 2 includes all possibilities, and we have assumed that the addition of two protons and one additional electron leads to cleavage of the O–O bond and dissociation of a water molecule, formally leaving an oxyl species, as shown in Fig. 2b. It is also possible that the catalytic cycle involves one additional reduction [15, 22, 25, 26], as indicated in Fig. 2c. If so, this implies that the resting-state Cu(II) is not part of the catalytic cycle; instead, the Cu(I) intermediate is formed after the rebound step, ready to bind O$_{2}$ in the next reaction cycle [25].

**Scheme 3:**
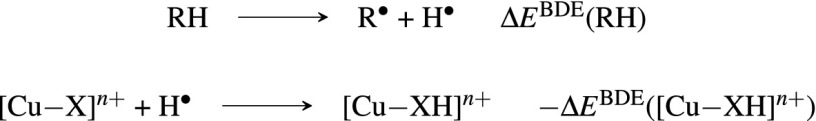
Homolytic bond cleavage reactions involving the intermediate and substrate

Only very few of the species in Fig. 2 have been investigated, viz. the superoxide complex, [Cu–OO]$^{+}$, and an axial isomer of a Cu(II)–oxyl species [22], [Cu–O]$^{+}$, has been considered in computational studies, but no other quantitative studies exist. A comprehensive computational study was performed by Abad et al. [27], but for another copper monooxygenase with a different active site. In studies of inorganic model systems, it has been suggested that a Cu(III)–hydroxy species could be responsible for the hydrogen-atom abstraction in the LPMOs [28–30]. In these studies, the feasibility of hydrogen-atom abstraction reactions of the type in Scheme 2 was estimated from bond-dissociation energies (BDEs) [28–36]. This is based on a formal separation of the reaction in Scheme 2 into the two reactions in Scheme 3.

Here, the first reaction is the homolytic R–H bond cleavage in RH and the second reaction is the reverse of the homolytic X–H bond cleavage of [Cu–XH]$^{n+}.$ Both reactions can be described by homolytic bond-dissociation energies, $\Delta E^{\text {BDE}}$(RH) and $\Delta E^{\text {BDE}}$([Cu-X]$^{n+}$), respectively. Consequently, we can describe the thermodynamics of the reaction in Scheme 1 by ${\Delta E^{\text {react}} = \Delta E^{\text {BDE}}(\mathrm {RH}) -\Delta E^{\text {BDE}}({\mathrm {[Cu{\hbox{--}}XH]}^{n+}})}$, and thus, the hydrogen-abstraction ability of a certain [Cu–X]$^{n+}$ complex is described by $\Delta E^{\text {BDE}}$([Cu–XH]$^{n+}$).

Unfortunately, experimental BDEs involve laborious measurements of p$K_\mathrm{a}$ values and reduction potentials [37–40] in strictly controlled environments. This is involved and time-consuming already for inorganic model systems and it becomes even more complicated for proteins. It is not certain that BDEs are transferable from model complexes to a protein active site, because the ligand spheres are typically quite different. With computational methods, BDEs are straightforwardly calculated and close mimics of the protein active site can be directly employed.

Based on a QM/MM-optimised structure for the Cu(II)–superoxide, [Cu–OO]$^{+}$, we will here investigate a number of putative reactive LPMO species, as shown in Fig. 2. Our aim is to identify putative intermediates with sufficiently high BDEs to activate the C$_1$–H or C$_4$–H bonds in polysaccharides. We will also investigate species in which the terminal NH$_{2}$ unit is deprotonated, as this has recently been suggested as a possible way to increase the BDE [20, 25, 30]. Finally, we also investigate whether the axial tyrosine ligand affects the BDE, because some LPMOs lack this ligand.

## Computational methods

### The starting structure

**Fig. 3:**
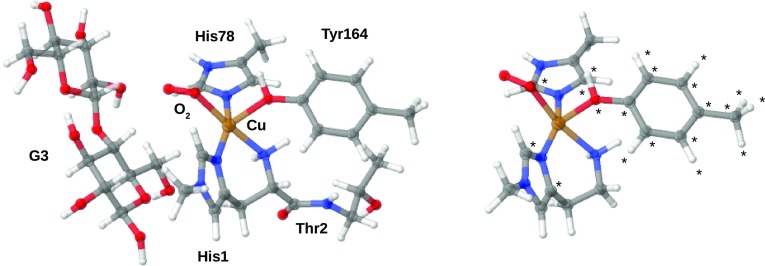
*Left* The QM systems employed in the QM/MM optimisation of [Cu–OO]$^{+}$. *Right* The truncated system employed for calculations of BDEs for [Cu–OO]$^{+}$. The other intermediates were truncated similarly, but with varying ligands (cf. Fig. 2). Atoms marked with “$\star$” were frozen in the structure optimisations. Color codes: Cu is *brown*, C is *gray*, O is *red*, N is *blue*, and H is *white*

The starting structures for the BDE calculations were obtained from a QM/MM optimisation of a LPMO–substrate (oligosaccharide) complex with the active site in the [Cu–OO]$^{+}$ triplet state. This QM/MM optimisation was started from a recent 1.8-Å resolution X-ray structure of an LPMO–substrate complex from *Lentinus similis* [20]. The QM region is shown in Fig. 3 (*left*). A more detailed account of this QM/MM optimisation will be given in a separate study regarding the full reaction mechanism of the enzyme.

### Bond-dissociation energies

$\Delta E^{\text {BDE}}$ was calculated according to Scheme 3. For the LPMO intermediates, this proceeds by truncation of the QM/MM-optimised structure: starting from the QM region, the substrate was deleted, as well as all atoms in Thr2 and the backbone O atom of His1, converting the C$^{\alpha }$ atom to a hydrogen. The truncated system is shown in Fig. 3 (*right*) for the [Cu–OO]$^{+}$ intermediate. To ensure that the active site does not change too much from the protein structure in the BDE calculations, we have frozen the two carbon atoms bound to the coordinating nitrogen atoms of the imidazole rings in the structure optimizations, cf. Fig. 3 (*right*). For the Tyr164 ligand, we have previously experienced large changes in Cu–O bond distances based on vacuum optimisations [23]. However, this ligand has been suggested to be involved in the mechanism [20], and we, therefore, decided to keep it, but with all atoms frozen to their position in the QM/MM-optimised structure. From this starting point, we optimised the structures of all the [Cu–X]$^{n+}$ species in the dashed boxes (a)–(c) in Fig. 2. We also optimised the corresponding product, [Cu–XH]$^{n+}$, of the second reaction of Scheme 3. Accordingly, for systems with an intact O–O bond, we optimised [Cu–OOH]$^{2+}$ and [Cu–OO]$^{+}$ as well as the products [Cu–OHOH]$^{2+}$ and [Cu–OOH]$^{+}$. The species in red color in Fig. 2 were not considered as reactants of the BDE reaction (but [Cu–OOH]$^{+}$ was considered as a product): the [Cu–O]$^{3+}$ and [Cu–OH]$^{4+}$ moieties correspond to a formal Cu(IV) oxidation state, which we deem unlikely. Moreover, attempts to optimise the reduced [Cu–OO]$^{0}$ and [Cu–OOH]$^{+}$ species gave rise to severely distorted structures, either for the reactant ([Cu–OO]) or for the hydrogenated products, [Cu–OOH] and [Cu–OHOH]$^{+}.$ The distortion of the structures is either due the fact that Cu(I) prefers a tetrahedral coordination environment or that the imidazole ligands are reduced instead of Cu(II). Removal of the tyrosine ligand in the hydrogenation of [Cu–OO]$^{0}$ led to a [Cu–(O)(OH)] complex, where the O–O bond was broken. The corresponding BDE was rather low; 260 kJ/mol with TPSS/def2-SV(P), showing that it is no competitive candidate for the reactive intermediate. In addition, we have previously shown for the resting state in QM/MM optimisations that the reduction to Cu(I) is problematic to study in vacuum [23]. The [Cu–OOH]$^{+}$ and [Cu–OO]$^{0}$ species are, therefore, not further considered.

As shown in Fig. 2b, an oxyl species, [Cu–O]$^{2+}$, can be formed from the [Cu–OO]$^{+}$ intermediate after reduction and a double protonation, leading to cleavage of the O–O bond and dissociation of H$_{2}$O. As discussed in the introduction, it is possible that the resting Cu(II) state is not involved in the actual catalytic cycle and that the putative intermediates are reduced one step further. Therefore, we also investigated the [Cu–O]$^{+}$ species, cf. Fig. 2c. The corresponding products of hydrogen-atom abstraction for [Cu–O]$^{2+}$ and [Cu–O]$^{+}$ are [Cu–OH]$^{2+}$ and [Cu–OH]$^{+}$. We also investigated the protonated forms of the two oxyl species, namely, [Cu–OH]$^{3+}$ and [Cu–OH]$^{2+}$, and the corresponding products of hydrogenation, [Cu–OH$_{2}$]$^{3+}$ and [Cu–OH$_{2}$]$^{2+}$.

Calculations on all the above [Cu–X]$^{n+}$ species were repeated with the terminal NH$_{2}$ group deprotonated. For three putative intermediates ([Cu–OO]$^{+}$, [Cu–O]$^{2+}$, and [Cu–O]$^{2+}$), we also studied the complexes with the OH group of tyrosine deprotonated.

To estimate the C–H bond-activation power of the investigated species, we compare the calculated BDEs with the BDEs of the substrate (this will also allow us to calculate $\Delta E^{\text {react}}$ in Scheme 1). LPMOs oxidise either the C$_{4}$–H or the C$_{1}$–H bonds in the polysaccharide, and we have therefore only calculated these BDEs. The BDEs for the C$_{4}$–H and C$_{1}$–H in the trisaccharide substrate were calculated by a similar procedure as for the [Cu–X]$^{n+}$ species: the structure optimisation was started from the QM/MM-optimised structure of the LPMO–substrate complex in the [Cu–OO]$^{+}$ intermediate. These optimisations were carried out without any frozen atoms and the structural changes were minor, compared to the QM/MM-optimised structure.

The computational protocol was similar to that in our QM/MM study of LPMO enzymes [23]: the structures were optimised with the def2-SV(P) basis set [41, 42] and the dispersion-corrected TPSS-D3 functional [43, 44] with Becke–Johnson damping [45]. With the sole exception of the species without the tyrosine ligand (see below), all calculations employed an implicit solvation model [46] (COSMO) with a dielectric constant ($\varepsilon$) of 4.0. Default radii where employed for all *p*-block atoms, whereas we employed a radius of 2.0 Å for Cu. The BDEs were calculated from these structures by single-point calculations with the def2-TZVPP and def2-QZVPP basis sets [41, 42]. The def2-QZVPP results are presented here, whereas results with the def2-SV(P) and def2-TZVPP basis sets are given in the supporting information (SI, Tables S1 and S2). The BDEs calculated with the def2-TZVPP and def2-QZVPP basis sets always agreed within 4 kJ/mol.

### Spin states

Many of the species in Fig. 2 and their hydrogenated products may attain several spin states. For example, [Cu–OH]$^{2+}$ can be interpreted as Cu(II) + OH$^{\cdot }$, i.e., with one unpaired electron on Cu and another on OH. These two unpaired spins can either be parallel, giving rise to a triplet spin state, or antiparallel, giving rise to an open-shell singlet. However, it can also be interpreted as Cu(III) + OH$^{-}$, which is a closed-shell singlet. Therefore, for species with even number of electrons, we studied all these three spin states (the open-shell singlet was studied with the broken-symmetry approach [47]). For species with an odd number of electrons, we only considered the doublet state. The singlet–triplet splittings are given in the SI, Tables S3–S5. All BDEs and reaction energies reported here are calculated with the respective electronic ground states.

### Role of the axial tyrosine ligand

We have also carried out a series of calculations on models without the tyrosine ligand. These calculations serve two purposes: first, they allow us to probe the influence of the axial tyrosine on the bond-activation power, which may be relevant to unravel weather LPMOs without this ligand employ a different mechanism. Second, they allow us to avoid any frozen atoms, and therefore, we could carry out a frequency analysis to estimate the thermal and entropic contributions [48] to $\Delta H^{\text {BDE}}_{T=298.15}$ and $\Delta G^{\text {BDE}}_{T=298.15}$. The thermoprogram [49] was employed to calculate $\Delta H^{\text {BDE}}_{T=298.15}$ and $\Delta G^{\text {BDE}}_{T=298.15}$.

### Effect of the chosen DFT functional

It is well known that the choice of DFT functional can affect calculated energetics, particularly for transition-metal systems as studied here. Therefore, we repeated all calculations for the species that included tyrosine with the B3LYP-D3 [50–52] functional and the def2-TZVPP basis sets. The result is included in Tables S1–S4. The change of functional does not alter any conclusions regarding which intermediates are most likely. However, it should be emphasized that both the effect on spin-state splittings and the calculated dissociation energies for the LPMO intermediates can be rather large, particularly for species with the O$_{2}^{-}$ or O$^{-}$ moieties protonated. For the C–H dissociation energies of the substrate, the effect is much smaller. Unless explicitly stated, results reported in the article were obtained with TPSS-D3 functional and def2-QZVPP basis sets.

### Calculation of reduction potentials and p$\varvec{K}_\mathrm{a}$ values

In an attempt to access the stability of the most interesting putative intermediates, we have (for these) calculated p$K_\mathrm{a}$ values and reduction potentials, $E^{o}$. The reduction potentials were calculated as absolute potentials and related to the standard hydrogen electrode by subtracting 4.28 V from the results [53]. Likewise, absolute p$K_\mathrm{a}$ values were obtained by subtracting 1131.00 kJ/mol from the difference in electronic energies of the protonated and deprotonated states. The value of 1131.00 kJ/mol represents the solvation free energy of H$^{+}$, the translational energy of the proton, as well as the change in the reference state from pressures to concentrations [53]. Calculated reduction potentials and p$K_\mathrm{a}$ values are very sensitive to the computational setup, including the description of the protein or solvent environment. When reporting these values, it should be remembered that we here represent the protein environment through a continuum model, which is a crude model for a protein; in particular, the dielectric constant is unknown and poorly defined for such an inhomogeneous system. Therefore, we probe sensitivity of the calculated p$K_\mathrm{a}$ values and reduction potentials using two different dielectric constants. Thus, in addition to the results with a dielectric constant of $\varepsilon =4.0$, we have also calculated p$K_\mathrm{a}$ values and reduction potentials with $\varepsilon =80.0$. Calculations with $\varepsilon =80.0$ were calculated as single-point calculations from the structures obtained with $\varepsilon =4.0$.

## Results

The calculated BDEs for the [Cu–XH]$^{n+}$ species and for the substrate are given in Table 1. It can be seen that for the [Cu–OOH]$^{2+}$ species, the BDE is rather low, 317 kJ/mol, compared to 423–434 kJ/mol for the saccharide substrate, which excludes this species as a putative reactive intermediate for C–H activation in LPMO. The same applies to the [Cu–OO]$^{+}$ intermediate, for which the BDE is 301 kJ/mol.

**Table 1:** Hydrogen homolytic BDEs (in kJ/mol) calculated for reactions involving the putative intermediates in Fig. 2 or the substrate

Complex	With Tyr164	Without Tyr164			
	$\Delta E^{\text {BDE}}$	$\Delta E^{\text {BDE}}$	ZPVE$^{\rm a}$	$\Delta H^{\text {BDE}}_{T=298.15}$ ^b^	$\Delta G^{\text {BDE}}_{T=298.15}$ ^b^
[Cu–OO]$^{+}$	301.3	300.7	−29.2	271.1	271.5
[Cu–OOH]$^{2+}$	317.3	323.6	−27.0	293.3	301.0
[Cu–O]$^{2+}$	467.9	466.3	−30.0	435.5	436.7
[Cu–OH]$^{3+}$	404.3	426.3	−28.4	394.2	402.4
[Cu–O]$^{+}$	458.8	458.0	−28.8	428.5	428.7
[Cu–OH]$^{2+}$	387.0	383.8	−25.7	353.8	363.8
Substrate	$\Delta E^{\text {BDE}}$		ZPVE^a^	$\Delta H^{\text {BDE}}_{T=298.15}$ ^b^	$\Delta G^{\text {BDE}}_{T=298.15}$ ^b^
C$_1$–H	422.5		−35.1	388.1	386.2
C$_4$–H	433.8		−34.8	399.1	398.9

All calculations were carried out with the COSMO continuum solvent ($\varepsilon =4.0$), the TPSS-D3 functional, and the def2-QZVPP basis set, based on structures optimised with TPSS-D3/def2-SV(P)

^a^ Zero-point vibrational energy from harmonic frequencies calculated with TPSS-D3/def2-SV(P) in vacuum

^b^Calculated from the harmonic frequencies with $\Delta E^{\text {BDE}}$ from def2-QZVPP single-point calculations on structures obtained with TPSS-D3/def2-SV(P), including the COSMO continuum solvent

If we move to the species in which the O–O bond has been cleaved, another picture emerges. For the [Cu–OH]$^{3+}$ species, the BDE is 404 kJ/mol, which indicates that it is more likely to perform the hydrogen abstraction, although the BDE is still slightly lower than that of the substrate. The copper–oxyl species, [Cu–O]$^{2+}$, is even more reactive and the BDE for a hydrogen-abstraction reaction involving this species is 468 kJ/mol, well above the BDEs of the substrate. These two species have not been investigated computationally before, but the former has been suggested based on studies on synthetic model complexes [30]. The present results indicate that both are promising candidates for the reactive intermediate. The corresponding reduced forms, [Cu–OH]$^{2+}$ and [Cu–O]$^{+}$, also give rise to high BDEs, 387 and 459 kJ/mol, respectively.

Table 1 also contains results calculated without the Tyr164 ligand. It can be seen that this ligand (as well as the constrained geometries) in general has a small effect on the calculated BDEs (1–6 kJ/mol), except in the case of [Cu–OH]$^{3+}$, for which the effect is around 20 kJ/mol. A closer analysis of the latter intermediate shows that the unpaired electron localizes on the tyrosine ligand. When this ligand is removed, the electron instead localizes on the imidazole ring of His1, leading to a destabilization of [Cu–OH]$^{3+}$, which in turn increases $\Delta E^{\text {BDE}}$.

Comparing $\Delta G^{\text {BDE}}_{T=298.15}$ and $\Delta H^{\text {BDE}}_{T=298.15}$ in Table 1 shows that the entropy plays only a minor role (less than 10 kJ/mol). Moreover, the difference between $\Delta E^{\text {BDE}}$ and $\Delta H^{\text {BDE}}_{T=298.15}$ is found to be 30–31 kJ/mol, which comes almost exclusively from the zero-point vibrational energy. However, these effect do not change the conclusion that the four complexes with a cleaved O–O bond all are putative candidates for the reactive intermediate of LPMO (and $\Delta G^{\text {BDE}}_{T=298.15}$ for [Cu–OH]$^{3+}$ is also larger than that of the substrate).

### Effect of deprotonation

Recently, the terminal amino group of the histidine brace have been proposed to play some role in the catalytic reaction. For instance, it participates in a hydrogen bonding network with the substrate [20] which increases the basicity and provides a channel for deprotonation of the N terminus. It has also been suggested to be deprotonated from studies on model complexes [30], and lately, indications have also come from neutron diffraction and X-ray crystallography [54]. Therefore, we also investigated the BDEs for reactions involving deprotonated complexes (i.e., involving –NH$^{-}$). The resulting BDEs are collected in Table 2. For three intermediates, we also investigated the effect of deprotonating the Tyr164 OH group.

**Table 2:** Hydrogen BDEs calculated for the intermediates in Fig. 2 with a deprotonated group, either the amino-terminal or the side chain of Tyr164

Complex	$\Delta E^{\text {BDE}}_{\text {Deprot.}}$	
	NH$^{-}$	O$^{-}$
[Cu–OOH]$^{2+}$	266.5	–
[Cu–OO]$^{+}$	302.4	297.9
[Cu–OH]$^{3+}$	387.6	
[Cu–O]$^{2+}$	468.7	467.5
[Cu–OH]$^{2+}$	308.3	
[Cu–O]$^{+}$	433.9	449.2

All calculations were carried out with the TPSS-D3 functional and the def2-TZVPP basis sets, based on structures optimised with TPSS-D3/def2-SV(P). They were performed in a COSMO continuum solvent with a dielectric constant of 4.0

In two cases, deprotonation of the amino-terminal has a large effect on the BDEs, namely, for [Cu–OH]$^{2+}$ and [Cu–OOH]$^{2+}$. For the former, the BDE is reduced by 79 kJ/mol, whereas for the latter, it decreases by 51 kJ/mol. In both cases, the change can be traced to the electronic structure of the product after hydrogen abstraction. A closer analysis of the electronic structures of these products shows that the deprotonation of the NH$_{2}$ group leads to increased spin-localization on the terminal NH-unit which destabilizes [Cu–OH$_{2}$]$^{2+}$ and [Cu–OHOH]$^{2+}$, thereby decreasing the BDE. This has also an influence on the molecular structures: for the [Cu–OH$_{2}$]$^{2+}$ species, the Cu–OH$_{2}$ bond is significantly elongated: the bond is 2.37 versus 2.08 Å in the form with the terminal amino group protonated, cf. Figs. S1 and S2. The water molecule is thus close to dissociation in the deprotonated form, although still weakly coordinated. A similar elongation is seen for [Cu–OHOH]$^{2+}$, where the deprotonated form has a Cu–O distance of 2.44 versus 2.16 Å in the protonated form. The corresponding distances in [Cu–OOH]$^{2+}$ are 1.93 Å (deprotonated) and 1.90 Å (protonated), cf. Figs. S3 and S4, respectively. For all the other intermediates, the deprotonation does not alter the BDEs markedly.

We also note that in two cases, deprotonation of the NH$_{2}$ group affects the spin-state splittings (see Tables S3 and S5). However, the spin-state splittings strongly dependent on the employed functional and should, therefore, be taken with some caution. For instance, for the [Cu–OH]$^{2+}$ species, the triplet state is stabilized. The stabilization occurs in connection with localization of excess spin on the NH ligand, similar to what was discussed above. For the TPSS functional, the singlet is still most stable in the deprotonated form, but with the B3LYP functional, the triplet state is most stable (as could be expected from the general stabilization of high-spin states with hybrid functionals).

The [Cu–O]$^{+}$ and [Cu–OH$_{2}$]$^{3+}$ species are even more delicate: in both cases, the splitting between the triplet state and the open-shell singlet is rather small for the form with NH$_{2}$ protonated, employing the TPSS functional. In [Cu–O]$^{+},$ the triplet is marginally more stable (2 kJ/mol), whereas the open-shell singlet is slightly more stable for [Cu–OH$_{2}$]$^{3+}$ (3 kJ/mol). However, when the NH$_{2}$ groups are deprotonated, the open-shell singlet is most stable by 25 kJ/mol for [Cu–O]$^{+}$, while the triplet is most stable (by 11 kJ/mol) for [Cu–OH$_{2}$]$^{3+}$. Again, the use of B3LYP alters these values both quantitatively and qualitatively. Clearly, DFT is not sufficiently accurate to determine the most stable spin state. We are currently investigate this matter with a more accurate method based on a multiconfigurational wave function.

Finally, deprotonation of the OH group has a small effect on the calculated BDEs. Interestingly, when comparing the complexes with deprotonated NH$_{2}$ and OH groups, respectively, the tyrosinate forms always have the lowest energy (by 40–110 kJ/mol), showing that it should be easier to deprotonate the tyrosine than the amine group, as could also be expected from the much higher p$K_\mathrm{a}$ value of the amino group, compared to tyrosine.

## Conclusions and discussion

In this paper, we have investigated the thermodynamic driving force for hydrogen abstraction, in terms of the BDE, for a number of possible intermediates of LPMO, as shown in Fig. 2. The calculations were started from a QM/MM structure of the substrate–LPMO complex with a bound superoxide, based on a recently published crystal structure [20]. Our calculated BDEs of the substrate are 434 kJ/mol for C$_{4}$–H and 423 kJ/mol for C$_{1}$–H. The corresponding Gibbs free energies are 399 and 386 kJ/mol, respectively. In comparison, a study of $\alpha$-1-*O*-methyl-D-glycopyranose with the B3LYP functional and the 6-311G++(p,d) basis set gave enthalpies of 388 and 393 kJ/mol, respectively, [55], i.e., close to our estimates. Comparing these numbers with the calculated BDEs of the putative LPMO intermediates, hydrogen abstraction for complexes with an intact O–O bond (i.e., involving Cu–O$_{2}$) is thermodynamically unfavorable. Using $\Delta E^{\text {BDE}}({\mathrm {C}_{4}-\mathrm {H}}$) in Table 1, the reactions involving [Cu–OOH]$^{2+}$ and [Cu–OO]$^{+}$ would give rise to endothermic reaction energies, $\Delta E^{\text {react}}$ (cf. Scheme 2), of 117 kJ/mol or 132 kJ/mol. The corresponding activation barriers would be even higher and would, therefore, give rise to too slow reactions compared to the experimental rate of 0.11 s$^{-1}$, which translate into a activation energy of approximately 60 kJ/mol [20].

However, for complexes in which the O–O bond is cleaved, the calculated BDEs are high and these intermediates seem to be more probable reactive intermediates. The reaction energies for the hydroxy complexes [Cu–OH]$^{2+}$ and [Cu–OH]$^{3+}$ are still somewhat endothermic, 47 and 29 kJ/mol, although the reaction energies becomes exothermic if the B3LYP functional is employed. The oxyl complexes [Cu–O]$^{+}$ and [Cu–O]$^{2+}$ always give rise to exothermic reaction energies: −25 and −34 kJ/mol (with the TPSS functional). This indicates that all these four states are possible intermediates in the reaction of LPMO.

The fact that our study suggests that O–O bond breaking occurs prior to hydrogen abstraction from the substrate is commensurate with recent experimental suggestions for the mechanism: the LPMO that we employed here for the QM/MM optimisation is known to only attack the C$_{4}$–H bond in the polysaccharide substrate. In a recent study on the same LPMO enzyme, O’Dell et al. [24] argued that this regio-selectivity only occurs if the oxygen atom directly bound to Cu is transferred, which is indeed the case if the O–O bond is broken before the hydrogen-atom abstraction.

In an attempt to obtain an approximate ranking of the four possible intermediates, we have compared calculated p$K_\mathrm{a}$ values and reduction potentials of the [Cu–X]$^{n+}$ moiety and p$K_\mathrm{a}$ value of the terminal NH$_{2}$ group (see SI, Tables S6 and S7). Unfortunately, the results strongly depend on the employed dielectric constant in the COSMO calculations, making it difficult to infer any general trends. The dependency of the environment description is a general problem of relating calculated reduction potentials and p$K_\mathrm{a}$ values to their experimental counterparts. In our study, the p$K_\mathrm{a}$ values showed too large variations with the dielectric constant to provide any guidance. The reduction potentials are somewhat more stable. From these numbers, it is clear that with reduction potentials between 2 and 4 V (depending on the dielectric constant), the [Cu–OH]$^{3+}$ intermediate is readily reduced, making this intermediate less likely. Likewise, the [Cu–O]$^{2+}$ state is also easily reduced, with a reduction potential of 1–2 V. However, given the qualitative nature of this discussion, this intermediate cannot be discarded, although the calculations indicate that [Cu–O]$^{+}$ is more likely than [Cu–O]$^{2+}$.

Our conclusions regarding the BDEs do not depend on weather; the axial tyrosine is included in the calculations or not, illustrating that this group is not required to explain the thermodynamics in the catalytic turnover of LPMOs. Only in the case of [Cu–OH]$^{3+}$ was a significant change seen, but the intermediate without the tyrosine residue was more reactive. The increased reactivity is mainly due to destabilization of [Cu–OH]$^{3+}$ which is a radical with the excess spin density localised on the tyrosine. Thus, the increased reactivity will most likely come at the expense of an increase in energy to generate [Cu–OH]$^{3+}$. Intriguingly, a number of structurally characterized LPMOs that mainly target C$_{1}$–H bonds lack this tyrosine, while LPMOs that target either only C$_{4}$–H or both C$_{1}$–H and C$_{1}$–H bonds often have it [56]. Our study shows that the regio-selectivity is not due to a enhanced reactivity induced by tyrosine, as tyrosine does not affect the BDEs for any of the investigated intermediates. The passive role of tyrosine in relation to the BDEs is also supported by the fact that some LPMOs have a phenylalanine in this position. Furthermore, site-directed mutagenesis of the axial tyrosine ligand in an LPMO has been shown to lead to only a slightly reduced activity [12] and not to deactivation. The role of tyrosine seems to be elsewhere, perhaps stabilization of radical species, possibly making one or more alternative reaction routes available.

We further investigated deprotonation of tyrosine, which in all cases led to only a minor change in the BDE. The same is not always true for deprotonation of the terminal amino group. In the cases where deprotonation had a large effect, it generally made the BDE lower and hence the hydrogen abstraction less favourable. Moreover, for the intermediates that seems most likely, deprotonation of NH$_{2}$ always had a rather small effect. If deprotonation occurs, its role, therefore, seems to be a stabilization of hydroxide or oxyl moieties of high formal charge. The deprotonation had a large effect on both reduction potentials and pK$_\mathrm{a}$ values. For example, the reduction potential of [Cu–OH]$^{3+}$ decreased from 4.2–2.0 to 2.8–1.5 V. Despite this large change, the [Cu–OH]$^{3+}$ intermediate is still readily reduced with NH$_{2}$ deprotonated. On the other hand, the reduction potential of the [Cu–O]$^{2+}$ intermediate decreases from 2.3–1.0 V to between −0.1 and −0.2 V when the terminal NH$_{2}$ was group deprotonated. This intermediates could, therefore, become more likely if the NH$_{2}$ group is deprotonated.

In summary, we can conclude that the four intermediates [Cu–OH]$^{3+}$ [Cu–OH]$^{2+}$, [Cu–O]$^{2+}$, and [Cu–O]$^{+}$ are all reasonable, based on their BDEs. Based on the reduction potential, [Cu–OH]$^{3+}$ does not seem plausible, while the [Cu–O]$^{2+}$ intermediate only appears to be plausible if the terminal NH$_{2}$ group is deprotonated. We currently investigate the three intermediates [Cu–OH]$^{2+}$, [Cu–O]$^{2+},$ and [Cu–O]$^{+}$ in a full QM/MM setup. Furthermore, our study has also indicated that computational work with DFT on the LPMO active site can be sensitive to the employed functional. We are, therefore, investigating selected intermediates with multireference methods.

## Electronic supplementary material

Below is the link to the electronic supplementary material.

Supplementary material 1 (pdf 436 KB)
