# Supplementary material for: Targeting the reactive intermediate in polysaccharide monooxygenases
Source: J Biol Inorg Chem. 2017 Jul 11;22(7):1029–37. doi: 10.1007/s00775-017-1480-1 (PMC5613103; doi:10.1007/s00775-017-1480-1)
Supplement: Supplementary file 1 — Supplementary material 1 (pdf 436 KB) [file 775_2017_1480_MOESM1_ESM.pdf]

Supporting information for: Targeting the Reactive Intermediate in Polysaccharide Monooxygenases

Erik D. Hedegård* and Ulf Ryde*

Department of Chemistry, Lund University, Kemicentrum Sölvegatan 39, Lund, Sweden

E-mail: erik.hedegard@teokem.lu.se; ulf.ryde@teokem.lu.se

For Peer Review

Table S1: Hydrogen bond dissociation energies in kJ/mol (kcal/mol). All calculations were carried out with the TPSS-D3 functional and based on structures optimised with TPSS-D3/def2-SV(P). All calculations were performed in a COSMO continuum solvent with a dielectric constant of 4.0.

**Complex**		TPSS-D3		B3LYP-D3
	def2-SV(P)	def2-TZVPP	def2-QZVPP	def2-TZVPP
$[Cu{-}OOH]^{2+}$	299.8 (71.7)	317.9 (76.0)	317.3 (75.8)	373.2 (89.2)
$[Cu{-}OO]^{+}$	264.2 (63.1)	300.8 (71.9)	301.5 (72.1)	311.9 (74.5)
$[Cu{-}OH]^{3+}$	406.8 (97.2)	406.1 (97.1)	404.3 (96.6)	463.0 (110.7)
$[Cu{-}O]^{2+}$	442.5 (105.8)	468.0 (111.9)	467.9 (111.8)	475.8 (113.7)
$[Cu{-}OH]^{2+}$	384.8 (92.0)	388.8 (92.9)	387.0 (92.5)	457.8 (109.4)
$[Cu{-}O]^{+}$	428.6 (102.4)	458.5 (109.6)	458.8 (109.7)	492.4 (117.7)
C1–H	417.0 (99.7)	422.9 (101.1)	422.5 (101.0)	424.5 (101.5)
C4–H	426.7 (102.0)	433.7 (103.7)	433.8 (103.7)	436.0 (104.2)

Table S2: Hydrogen bond dissociation energies in kJ/mol (kcal/mol) with the terminal NH_2 group deprotonated. All calculations were carried out with the TPSS-D3 functional and based on structures optimised with TPSS-D3/def2-SV(P). All calculations were performed in a COSMO continuum solvent with a dielectric constant of 4.0.

**Complex**		TPSS-D3		B3LYP-D3
	def2-SV(P)	def2-TZVPP	def2-QZVPP	def2-TZVPP
$[Cu{-}OOH]^{2+}$	251.0 (60.0)	267.6 (60.0)	266.5 (64.0)	324.3 (77.5)
$[Cu{-}OO]^{+}$	270.1 (64.6)	302.4 (72.3)	302.4 (72.2)	309.1 (73.9)
$[Cu{-}OH]^{3+}$	383.1 (91.6)	389.2 (93.0)	387.6 (92.6)	475.3 (113.6)
$[Cu{-}O]^{2+}$	439.3 (105.0)	470.2 (112.4)	471.0 (112.6)	473.5 (113.2)
$[Cu{-}OH]^{2+}$	303.9 (72.6)	310.2 (74.1)	308.3 (73.7)	284.3 (69.0)
$[Cu{-}O]^{+}$	424.3 (101.4)	435.6 (104.1)	433.9 (103.7)	467.4 (111.7)
C1–H	417.0 (99.7)	422.9 (101.1)	422.5 (101.0)	424.5 (101.5)
C4–H	426.7 (102.0)	433.7 (103.7)	433.8 (103.7)	436.0 (104.2)

For Peer Review

1 Singlet triplet splittings

Table S3: Singlet–triplet splitting, $\Delta E = E_T - E_S$ kJ/mol (kcal/mol), with Tyr164 included. Structures were obtained with TPSS-D3/def2-SV(P). All calculations were performed in a COSMO continuum solvent with a dielectric constant of 4.0.

**Complex**	TPSS-D3			B3LYP-D3
	def2-SV(P)	def2-TZVPP	def2-QZVPP	def2-TZVPP
$[Cu{-}OOH]^{2+a}$	18.9 (4.5)	27.7 (6.6)	27.7 (6.6)	-20.6 (-4.9)
$[Cu{-}OH_2]^{3+b}$	-28.8 (-6.4)	-26.7 (-6.4)	-26.5 (-6.3)	-87.2 (-20.9)
$[Cu{-}OH]^{2+a}$	41.3 (9.9)	52.8 (12.6)	53.7 (12.8)	33.8 (8.1)
$[Cu{-}OO]^{+b}$	-14.5 (-3.5)	-13.4 (-3.2)	-13.3 (-3.2)	-15.7 (-3.8)
$[Cu{-}O]^{+b}$	-16.8 (-4.0)	-7.1 (-1.7)	-5.8 (-1.4)	-25.0 (-6.0)

[a] Open-shell singlet either identical or higher in energy than the closed shell singlet.
[b] Calculated as open-shell singlet.

Table S4: Singlet–triplet splitting, $\Delta E = E_T - E_S$ kJ/mol (kcal/mol), without Tyr164. Structures were obtained with TPSS-D3/def2-SV(P). All calculations were performed in a COSMO continuum solvent with a dielectric constant of 4.0.

**Complex**	TPSS-D3		
	def2-SV(P)	def2-TZVPP	def2-QZVPP
$[Cu{-}OOH]^{2+a}$	19.0 (4.6)	30.6 (7.3)	31.8 (7.6)
$[Cu{-}OH_2]^{3+}$	-11.1 (-2.7)	-5.8 (-1.4)	-5.1 (-1.2)
$[Cu{-}OH]^{2+b}$	17.9 (4.3)	42.9 (10.3)	43.9 (10.5)
$[Cu{-}OO]^{+b}$	-15.1 (-3.6)	-14.1 (-3.4)	-14.9 (-3.6)
$[Cu{-}O]^{+b}$	-13.4 (-3.2)	-3.2 (-0.8)	-1.8 (-0.4)

[a] Open-shell singlet either identical or higher in energy than the closed shell singlet.
[b] Calculated as open-shell singlet.

For Peer Review

Table S5: Singlet–triplet splitting, $\Delta E = E_T - E_S$ kJ/mol (kcal/mol). Structures were obtained with TPSS-D3/def2-SV(P). All calculations were performed in a COSMO continuum solvent with a dielectric constant of 4.0.

**Complex**	Deprotonated	def2-SV(P)	def2-TZVPP	def2-QZVPP
$[Cu{-}OOH]^{2+a}$	NH_2	28.1 (6.7)	37.9 (9.1)	38.7 (9.2)
$[Cu{-}OH_2]^{3+a}$	NH_2	-10.9 (-2.6)	-3.4 (-0.8)	-2.83 (-0.7)
$[Cu{-}OH]^{2+b}$	NH_2	19.9 (4.8)	29.4 (7.0)	29.9 (7.2)
$[Cu{-}OO]^{+b}$	NH_2	-6.5 (-1.6)	-13.9 (-3.3)	-13.2 (-3.2)
$[Cu{-}O]^{+b}$	NH_2	3.2 (0.8)	23.2 (5.6)	25.3 (6.1)

[a] Open-shell singlet either identical or higher in energy than the closed shell singlet.
[b] Calculated as open-shell singlet.

For Peer Review

2 Reduction potentials and pK_a values

Table S6: Calculated reduction potentials (V) and pK_a values. Values for the species which the terminal NH_2 group deprotonated are also shown. All values are calculated the TPSS-D3 functional and the def2-TZVQPP basis sets, based on structures optimised with TPSS-D3/def2-SV(P) in a COSMO continuum solvent with a dielectric constant of 4.0.

**Complex**	E^o	E^o	E^o	E^o
	$\epsilon = 4.0$		$\epsilon = 80.0$	
	NH_2	NH	NH_2	NH
$[Cu{-}OH]^{3+}$	4.2	2.8	2.0	1.5
$[Cu{-}O]^{2+}$	2.3	-0.2	1.0	-0.1
**Complex**	pK_a	pK_a	pK_a	pK_a
$[Cu{-}OH]^{3+}$	-30	-6	8	16
$[Cu{-}OH]^{2+}$	2	40	25	43

Table S7: Calculated pK_a values for the terminal NH_2 group. All values are calculated the TPSS-D3 functional and the def2-TZVQPP basis sets, based on structures optimised with TPSS-D3/def2-SV(P). A COSMO continuum solvent was employed.

**Complex**	pK_a $\epsilon = 4.0$	pK_a $\epsilon = 80.0$
$[Cu{-}OH]^{3+}$	-33	5
$[Cu{-}OH]^{2+}$	-10	13
$[Cu{-}O]^{2+}$	-9	13
$[Cu{-}O]^{+}$	28	32

3 Structures

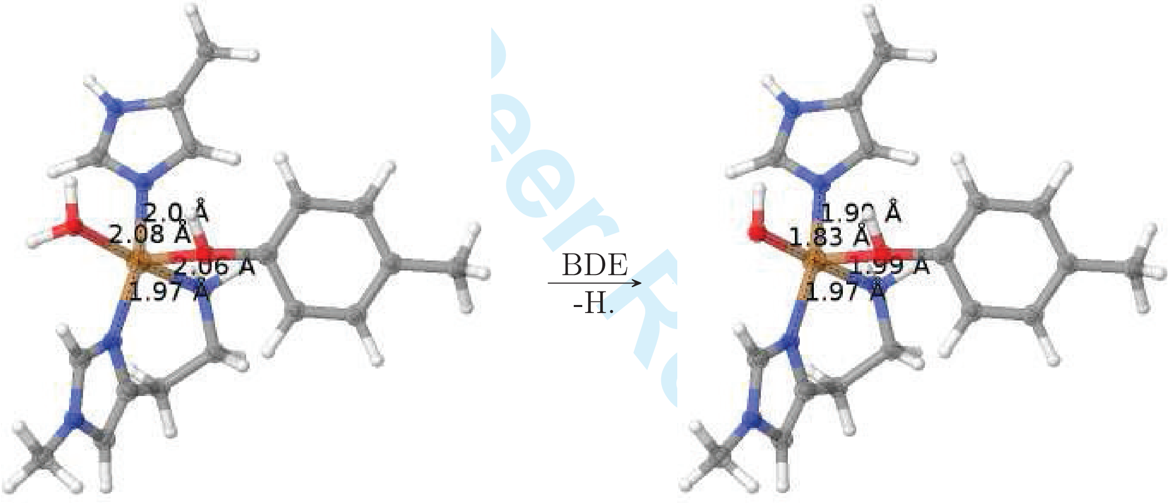

$[Cu{-}OH_2]^{2+}$ $[Cu{-}OH]^{2+}$

Figure S1: Optimised strucures of $[Cu{-}OH_2]^{2+}$ (S=$\frac{1}{2}$) and $[Cu{-}OH]^{2+}$ (S = 0) for calculation of BDE of $[Cu{-}OH_2]^{+}$.

For Peer Review

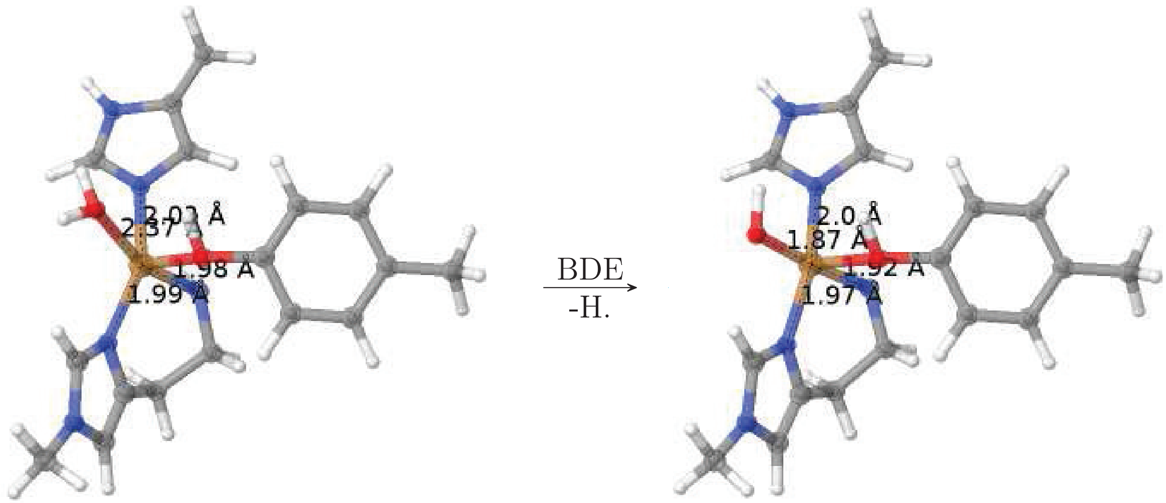

$[Cu{-}OH_2]^+$ $[Cu{-}OH]^+$

Figure S2: Optimised strucures of $[Cu{-}OH_2]^+$ ($S{=}\frac{1}{2}$) and $[Cu{-}OH]^+$ ($S = 0$) for calculation of BDE of $[Cu{-}OH_2]^+$ with the terminal NH_2 group deprotonated.

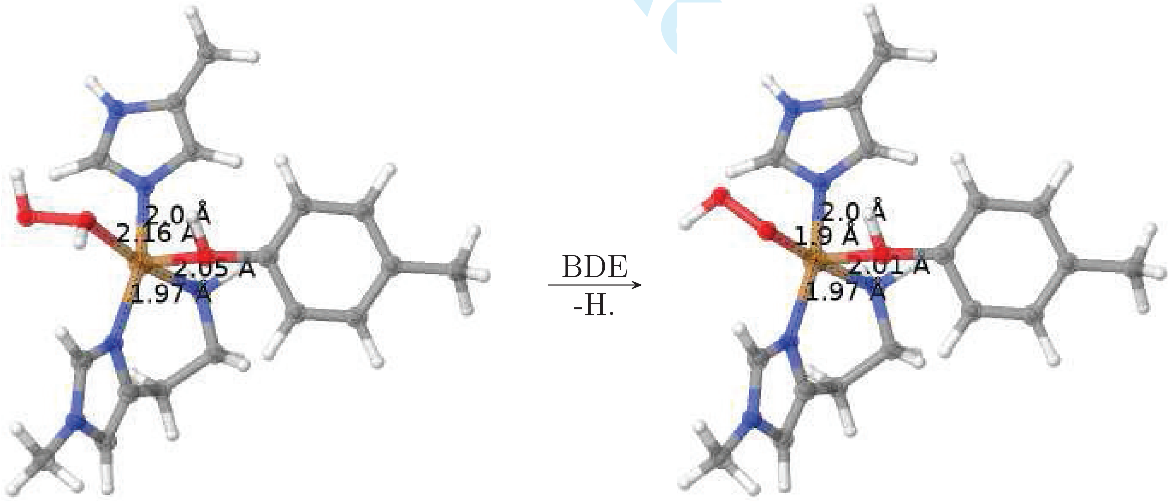

$[Cu{-}OHOH]^{2+}$ $[Cu{-}OOH]^{2+}$

Figure S3: Optimised structures of $[Cu{-}OHOH]^{2+}$ ($S{=}\frac{1}{2}$) and $[Cu{-}OOH]^{2+}$ ($S = 0$) for calculation of BDE of $[Cu{-}OOH]^{2+}$.

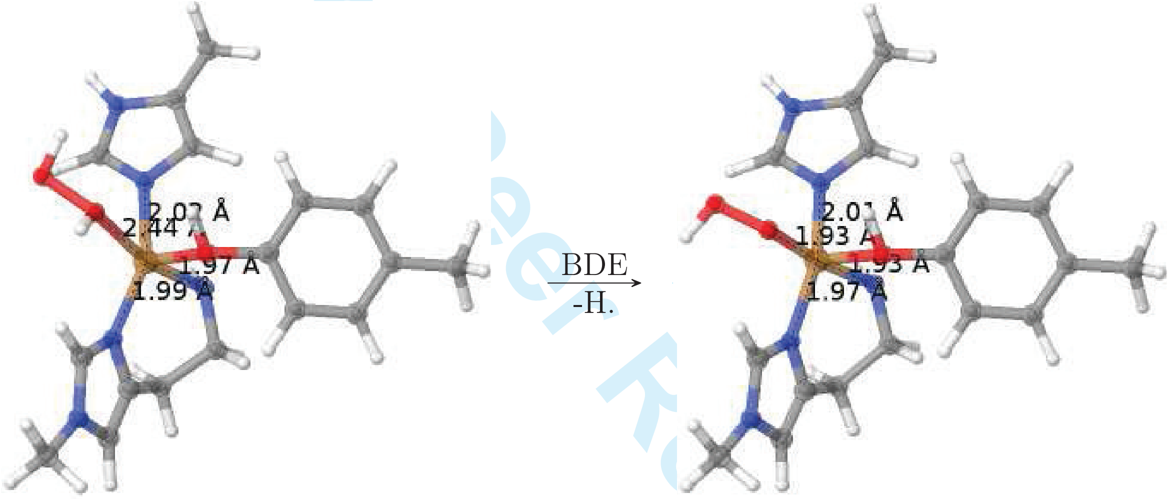

[Cu–OHOH]$^{2+}$ [Cu–OOH]$^{2+}$

Figure S4: Optimised structures of [Cu–OHOH]$^{2+}$ (S=$\frac{1}{2}$) and [Cu–OOH]$^{2+}$ (S = 0) for calculation of BDE of [Cu–OOH]$^{2+}$ with the terminal NH_2 group deprotonated.

For Peer Review
